# Mucosal Interleukin‐10 depletion in steroid‐refractory Crohn's disease patients

**DOI:** 10.1002/iid3.710

**Published:** 2022-09-27

**Authors:** Anna Carrasco, Eva Tristán, Fernando Fernández‐Bañares, Albert Martín‐Cardona, Montserrat Aceituno, Yamile Zabana, Lourdes Fluvià, José María Hernández, Violeta Lorén, Josep Manyé, Antonio Salas, Xavier Andújar, Carme Loras, Maria Esteve

**Affiliations:** ^1^ Department of Gastroenterology, Hospital Universitari Mútua Terrassa Universitat de Barcelona Catalonia Spain; ^2^ Centro de Investigación Biomédica en Red de Enfermedades Hepáticas y Digestivas (CIBERehd) Madrid Spain; ^3^ Proteomics and Metabolomics Core Facility, Institut de Recerca Germans Trias i Pujol (IGTP) Badalona Spain; ^4^ Research Group in Inflammatory Bowel Diseases, Institut de Recerca Germans Trias i Pujol (IGTP) Badalona Spain; ^5^ Department of Pathology Hospital Universitari Mutua Terrassa, Universitat de Barcelona Catalonia Spain

**Keywords:** Crohn's disease, inflammatory bowel disease, Interleukin‐10, intestinal compartmentalization, steroid resistance

## Abstract

**Background:**

Previous studies suggested that Interleukin‐10 (IL‐10) depletion in Crohn's disease (CD) could predict outcome. Aim: To determine IL‐10 in blood and at different intestinal locations in patients with active CD and to assess its potential prognostic capacity to identify aggressive CD.

**Methods:**

Twenty‐three patients with CD were included. Ulcerative colitis (UC), infectious colitis and healthy individuals acted as controls. Serum and mucosal samples were taken at baseline and 1 month after steroid initiation in CD patients. Patients were classified according to steroid response. Control samples were obtained from different intestinal locations. IL‐10 expression was measured with real‐time polymerase chain reaction, immunofluorescence (intestine) and ELISA (serum, biopsy cultures' supernatants and tissue homogenates).

**Results:**

CD and UC showed an increase in IL‐10 messenger RNA (mRNA) versus controls (*p* < .0001) in mucosa, whereas IL‐10 protein secretion was increased in all types of intestinal inflammation (*p* < .001). No differences in IL‐10 mRNA were found in CD at baseline regarding steroid response, but levels decreased in non‐responders versus responders (*p* = .027) and were restored with rescue therapy. Serum IL‐10 was increased in steroid‐refractory CD at baseline and after treatment.

**Conclusions:**

Abnormal IL‐10 levels in refractory patients in both mucosa and blood have physiopathological relevance and may have potential clinical applications.

## Graphical abstract

(1) Increased messenger RNA and protein lL‐10 levels in intestinal mucosa is a shared immunological response of both active Crohn's disease (CD) and ulcerative colitis. (2) CD patients showed a particular Interleukin‐10 (IL‐10) pattern related to steroid response, with depletion of IL‐10 at mucosal compartment in steroid resistant patients and restoration after successful rescue therapy. (3) At peripheral blood, high levels of circulating IL‐10 were found in steroid‐resistant CD patients before and after treatment initiation, suggesting potential clinical applications of IL‐10 as predictor of treatment response.

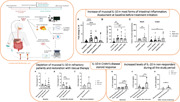

## 1 INTRODUCTION

Interleukin‐10 (IL‐10) plays a significant role in orchestrating intestinal immune homeostasis, and it is expressed by several cells of the innate and adaptive immune system.^1^ Many studies highlight the importance of the anti‐inflammatory IL‐10 axis in the physiopathology of inflammatory bowel disease (IBD). Genetic defects in the IL‐10/IL‐10‐receptor axis result in a very early severe, and sometimes fatal, form of IBD, supporting the hypothesis that IBD is mediated, at least in part, by an impaired immune system unable to control inflammatory mechanisms against microbiota.^2^

Despite the appealing therapeutic potential of IL‐10, clinical trials assessing its therapeutic efficacy in Crohn's Disease (CD) have been disappointing to date, probably due to the very short half‐life.^3^, ^4^

One potential explanation for this is the presence of individual differences among patients in disease severity, phenotype, and pretreatment IL‐10 levels.^3^ In fact, some studies have revealed that measuring differential baseline levels of IL‐10 in the mucosa might be useful to predict response to steroid treatment,^5^ evolution towards severe phenotypes (stricturing or penetrating),^6^ or early postoperative recurrence.^7^ Similarly, success in granulocyte‐colony stimulating factor treatment in CD patients is associated with an increase in peripheral IL‐10 secreting CD4^+^ memory T cells, whereas levels in nonresponder patients remained unchanged.^8^ Thus, evidence suggests that individual IL‐10 levels could be used in clinical practice to predict disease outcome, determining the need for immunosuppresants and biologics earlier in the disease course. Furthermore, the intestine comprises different functional and anatomic compartments that possess different, and unique, immune environments.^9^ This specialized compartment differentiation should be taken into account, especially when assessing immunological features of CD^10^ that might affect any region of the digestive tract.

We aimed to determine IL‐10 levels in patients with CD receiving conventional step‐care therapy and to assess the potential prognostic capacity of IL‐10 levels to identify aggressive CD (steroid‐refractory or steroid‐dependent and requiring immunosuppressants for disease control). To ascertain if potential changes in IL‐10 are related to the intestinal location or if they are shared with other types of intestinal inflammation IL‐10 levels will be assessed in different intestinal compartments and in peripheral blood, in healthy individuals and in patients with other types of intestinal inflammation (ulcerative colitis [UC] and infectious colitis [IC]).

## 2 MATERIAL AND METHODS

### 2.1 Patients and controls

Twenty‐three patients with active CD were included (age 36 ± 16 years old, 13 females). Patients with different types of intestinal inflammation were also included as a disease control groups: (1) active UC (*n* = 7; age 43 ± 14 years old, 3 females), and (2) IC (*n* = 5; age 32 ± 12 years old, 4 females), representative of IBD and non‐IBD intestinal inflammation, respectively.

The diagnosis of CD and UC was based on established diagnostic criteria.^11^, ^12^ Active disease was clinically defined in CD by CD Activity Index (CDAI)^13^ ≧ 150 and endoscopic activity by Simple Endoscopic Score for CD (SES‐CD) partial score (ranging from 0 to 3).^14^ In CD patients, samples were taken at baseline before steroid initiation and 1 month after starting steroids. Patients were classified as steroid sensitive, dependent, or refractory based on established clinical criteria.^12^ In all steroid‐refractory and ‐dependent patients a rescue therapy (medical or surgical) was necessary either immediately (in steroid‐refractory) or during the midterm follow‐up (steroid‐dependent). The appropriate classification with respect to steroid response was obtained 6 months after inclusion in the study for the steroid‐dependent group and after a maximum of 1 month in refractory patients. In nonresponder CD patients, samples were additionally taken after successful rescue medical therapy. In UC, active disease was defined as a Mayo score ≧ 4 with an endoscopic subscore ≧ 2.^15^ IC patients had sudden onset, acute diarrhea, fever, and positive inflammatory markers at the time of inclusion. The etiological agents were *Salmonella enteritidis* in two cases, *Clostridium difficile* in one case, *Campylobacter jejuni* in one case, and one case of self‐limited colitis with negative culture. In UC and IC patients, samples were taken only at baseline and before treatment initiation.

Ten control subjects (6 females, 48.3 ± 9.6 years) in whom colonoscopy was indicated for abdominal pain (*n* = 2) or colon cancer screening of the general population (*n* = 8) yielding a normal mucosa were also included. In controls, biopsies were obtained from three separate regions (ileum, right colon, and left colon) in a paired manner. Two biopsies from each segment were analyzed by an expert pathologist (A. S.) to ensure normality at the microscopic level.

Not all the samples were used in all the experiments. Specific sample size for each experiment is specified in the figure legends.

### 2.2 RNA and protein extraction

Two biopsies were collected in ice‐chilled RNA later (Ambion), stored for 24 h at 4°C, and then dry‐stored at −80° until use for simultaneous extraction of RNA and proteins. Dry biopsies were weighed and homogenized in QIAzol reagent (QIAgen) using gentleMACS tissue dissociator (RNA program, Milteny Biotech). A second dissociation round was applied if tissue fragments were still present. Chloroform was added and the mixture centrifuged, allowing for the separation of three different phases (upper aqueous phase containing RNA, white interphase containing DNA, and lower organic phase containing proteins and remaining tissue). The upper aqueous phase was separated and used for RNA extraction using miRNeasy Mini Kit (QIAgen) following the manufacturer's instructions. The lower organic phase was used for protein extraction by mixing it with absolute ethanol and then centrifuging (20 min, 2000*g*). Acetone was added to the supernatant which was incubated overnight at −20°C. After centrifugation, the pellet was allowed to dry at room temperature before adding 10 M urea, 5 mM DTT buffer for 1 h. The protein mixture was incubated at 95°C for 1 min, and then centrifuged, and the aliquots of the supernatant were stored at −80° until use.^16^

### 2.3 IL‐10 gene expression (real‐time **polymerase chain reaction** [PCR])

Primers and probes for IL‐10 (ref. Hs.PT.53a.2807216) and HPRT as a reference gene (ref. Hs.PT.58v.46521572) (both from Integrated DNA Technologies) were used in a 7300 PCR System (Applied Biosystems) using the Premix Ex Taq (TAKARA) as recommended by the manufacturers. All reactions were carried out in triplicate. Relative values (expressed as fold change) were calculated with the $2^{-\Delta \Delta C_{t}}$ method.^17^

### 2.4 IL‐10 protein (ELISA)

Protein levels were measured in serum, tissue homogenates (see above) and biopsy culture supernatants. For biopsy culture supernatants, two freshly isolated biopsies of similar size were immediately placed on a Petri dish containing 3 ml of Advanced RPMI culture media supplemented with antibiotics and 2% of FBS (all from GIBCO; Thermo Fisher Scientific) and cultured for 24 h at 37° and 5% CO_2_. Serum was separated by centrifugation (1300*g*, 10 min) from whole blood collected in BD Vacutainer tubes (SST‐II Advance; ref 366468; BD biosciences) and stored at −80 until use. IL‐10 ELISA kits were purchased from Millipore. All assays were performed in duplicate following the manufacturers’ instructions.

### 2.5 IL‐10 immunostaining (immunofluorescence)

Two biopsies were embedded in Tissue‐Tek OCT compound (Sakura; Finetek) and snap‐frozen in liquid nitrogen before transfer to −80°C for storage until use. Five micro meter sections were fixed with 4% paraformaldehyde for 2 h at room temperature. Triton‐X 0.4% was used to permeabilize for 15 min, and then 6% BSA solution was applied for 45 min. Overnight incubation with anti‐IL10 antibody (dilution 1:50; clone sc‐8438; Santa Cruz Biotechnology) was performed at RT in a humid chamber. Secondary antibody (goat‐anti‐mouse Alexa Fluor 594; 1:300 dilution; Lifetech) was then applied for 1 h. Hoescht (1:500 dilution; Lifetech) was used for nucleus counterstaining before mounting with Fluorescent Mounting medium (Dako). Slides were analyzed with an epifluorescence LeicaDMI6000B microscope and LeicaLAS‐AF software (provided by Advanced Optical Microscopy Department; University of Barcelona).

### 2.6 Image analysis

Images of all the available tissue were recorded (TileScan). For image analysis, 2 independent approaches (qualitative and quantitative) were taken. For qualitative quantification, we established a staining score according to intensity and distribution of IL‐10 staining for the subepithelial domain (SD) and for the rest of the lamina propria (LP) (Supporting Information: Figure 5). For quantitative analysis, ImageJ software was used. First, a Gaussian blur filter (radius of 1.00 pixel) was applied. Intensity of IL‐10 staining was calculated and the percentage of surface tissue stained was calculated over the nucleus surface.

### 2.7 Statistical analysis

Measures are expressed as mean ± standard error or median (interquartile range [IQR]). Nonparametric tests (Kruskall−Wallis test, Mann−Whitney *U* test, and Friedman test) for paired and unpaired data were used. The specific test applied for each comparison is detailed in the figure legends, and *p* < .05 was considered statistically significant. Statistical analysis was performed using IBM‐SPSS for Windows. Figures were made using GraphPad prism (Version 9.1.1).

## 3 RESULTS

### 3.1 Clinical characteristics of CD patients

Fifteen out of 23 CD patients had newly diagnosed disease and 8 had been previously diagnosed (years from diagnosis: median 3 years, range 1–6). Median CDAI at inclusion was 205.4 (IQR: 179.4−296.25) and median SES‐CD at the site of biopsy was three (IQR: 1−3). The disease was located at the ileum in nine patients, colon in nine, and ileum‐colon in the remaining five cases. Seven cases were steroid‐sensitive, eight steroid‐refractory, and eight steroid‐dependent. Sensitive patients had sustained remission for more than 1 year. All the patients were *naïve* for immunosuppressants and biologics at the time of inclusion.

### 3.2 IL‐10 expression in different intestinal compartments of healthy and inflamed bowel

**Figure 1:**
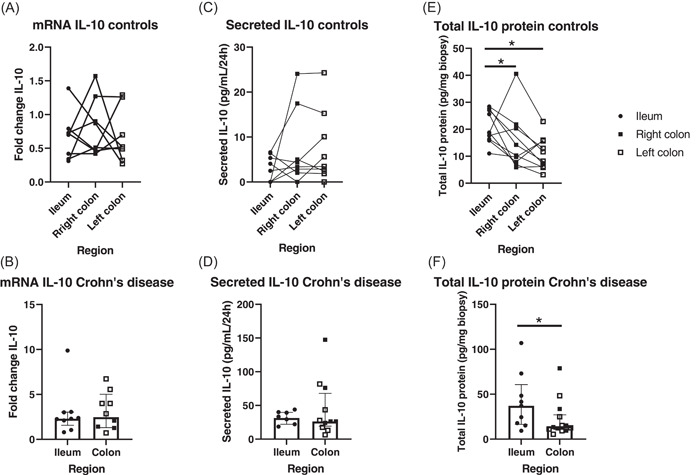
Interleukin‐10 (IL‐10) expression in different intestinal regions (ileum, right, and left colon) of controls (A, C, E) (*n* = 10 paired samples) and inflamed mucosa of patients with Crohn's disease (B, D, F) (*n* = 9 ileal and *n* = 12 colonic, non‐paired samples). Gene expression (A, B); secreted protein levels in culture supernatant of mucosal explants (C, D) and total protein of tissue homogenates (E, F). Line graph shows trends in data for the different intestinal regions. Bar graph represents median values and whiskers above and below 5%−95% percentile. Friedman Test (A, C, E); Mann−Whitney *U* Test (B, D, F). *p < .05. In (B) measurements are expressed as fold change over the samples of controls.

We first determined whether there were any differences related to intestinal region (ileum, right colon, and left colon). Regarding IL‐10 gene expression, no differences were found related to location in healthy bowel (*p* = .974, Figure 1A) or in inflamed samples from CD patients (*p* = .695, Figure 1B).

IL‐10 dynamics were further explored by measuring IL‐10 protein expression in culture supernatant of mucosal explants and in tissue homogenates. There were also no significant differences regarding the IL‐10 protein secreted by the culture explants between ileum, right colon, or left colon of the controls. However, half (5 out 10) of the ileal samples did not secrete enough protein to reach the lower limit for detection with our kit, whereas this happened in 3 out of 10 from the right and only 2 out of 10 samples from the left colon, resulting in a tendency to higher secretion of IL‐10 in colon as compared to ileum (*p* = .135, Figure 1C). Similarly, no differences related to location were found between ileal and colon samples of CD patients (*p* = .678, Figure 1D).

In contrast to IL‐10 gene expression and IL‐10 protein secreted in biopsy culture, region‐specific differences were found when evaluating total protein levels in tissue homogenates for both controls and inflamed bowel of CD patients. Ileum IL‐10 protein levels of controls were significantly increased when compared to their colonic counterparts (*p* = .025 vs. right colon and *p* = .028 vs. left colon, Figure 1E), whereas no differences were found between right and left colon samples (*p* = .401). This regional differentiation was also maintained in inflamed CD samples, in which ileal IL‐10 levels were significantly increased as compared to the colonic ones (*p* = .036 ileum vs. colon, Figure 1F), whereas comparable amounts of IL‐10 were found in right and left colon samples (*p* = .731, data not shown). Therefore, our results seem to point to a region‐dependent regulation of IL‐10 values at the protein—but not the mRNA—level in both healthy and CD intestinal mucosa. This is of critical importance, especially when considering assessment of immunological differences in CD samples. Hence, for the subsequent comparisons of inflamed and healthy biopsies at the protein but not the mRNA IL‐10 level, we only used values obtained from the same region, either ileum or colon.

Taking into account the differences of the tissue homogenate in IL‐10 protein, we aimed to determine whether these differences could be monitored and measured through immunofluorescence staining analysis. We also aimed to uncover the cell morphology responsible for IL‐10 production and location of the positive stained cells. In this sense, we were unable to find significant differences in median fluorescence intensity (MFI) or type and cell distribution of positive Il‐10 cells in LP or SD between different intestinal compartments, either in healthy or CD‐inflamed intestine (Supporting Information: Figure 1).

### 3.3 Mucosal IL‐10 expression in different forms of intestinal inflammation

**Figure 2:**
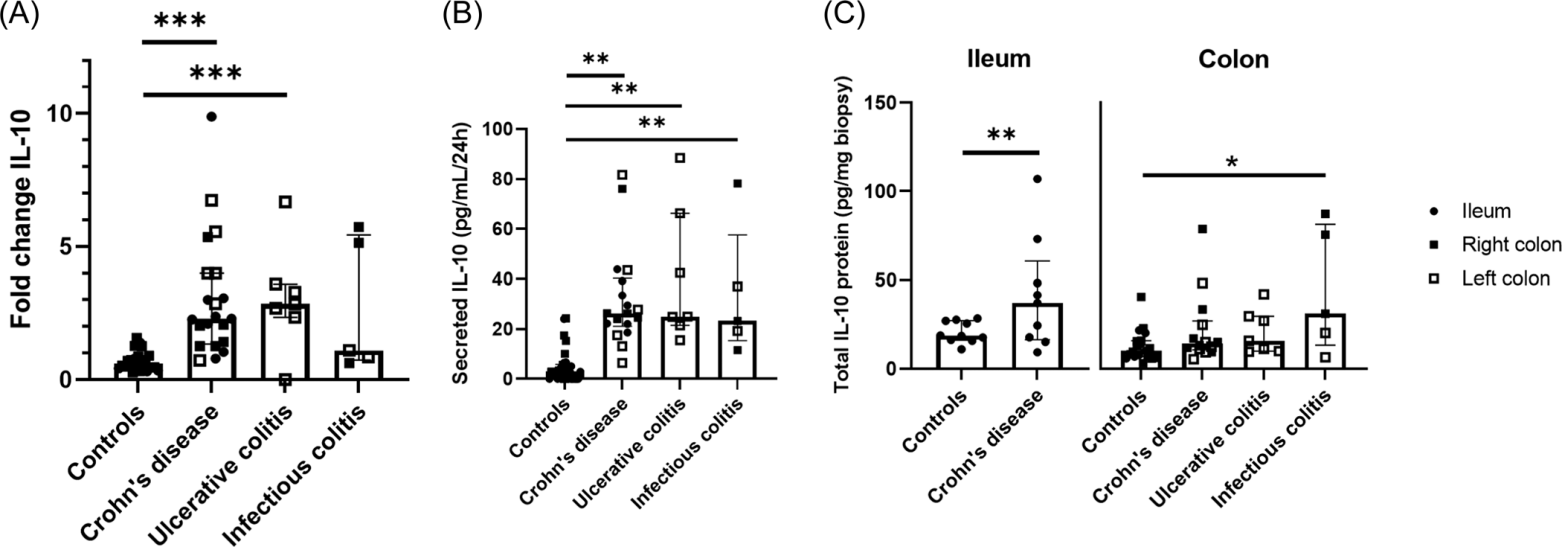
Interleukin‐10 (IL‐10) expression in controls (*n* = 24), Crohn's disease (*n* = 21), ulcerative colitis (*n* = 7), and infectious colitis (*n* = 5) (Gene expression [A], secreted protein expression in culture supernatant of mucosal explants [B] and total protein of tissue homogenates [C]). Bar graph represents median values and whiskers above and below 5%−95% percentile. Kruskall−Wallis Test. *p < .05 versus controls; **p < .005 versus controls; ***p < .0005 versus controls.

Both IBD forms of chronic intestinal inflammation, CD and UC, but not IC, showed a significant and similar increase in IL‐10 mRNA levels as compared to controls (*p* < .0001). The great dispersion of the results found for infectious colitis probably reflects diverse etiological agents, making it difficult to find significant differences in comparison to controls (Figure 2A). In the same way, similar behavior was found regarding IL‐10 protein secretion in culture supernatant. In this case, all forms of intestinal inflammation, including IC, showed a dramatic increase in secreted IL‐10 compared to controls (*p* < .001) (Figure 2B).

The assessment of IL‐10 protein expression in tissue homogenate was evaluated separately in ileum and colon because, as has been shown, increased levels in healthy ileal mucosa in comparison to colon were found. A significant increase in IL‐10 protein expression was found for infectious colitis compared to colonic mucosa in controls (*p* = .050), but not for colonic CD (*p* = .22) or for UC (*p* = .14). By contrast, IL‐10 protein levels in ileal CD showed a significant increase compared to healthy ileum (*p* = .050) (Figure 2C). Nor did IL‐10 protein expression assessed with immunofluorescence show disease‐related differences (CD, UC, IC vs. controls) (Supporting Information: Figure 1). However, we recognized positive IL‐10 cells located in the SD with dendritic cell morphology—considered to be one of the main sources of IL‐10 in the intestine.^1^ But in addition, we also identified considerable amounts of IL‐10 positive mononuclear cell infiltration in the LP irrespective of the diagnose, or intestinal location, in both healthy and inflamed tissue. See Supporting Information: Figure 5 for overview of the scoring system used and representative images obtained.

We also assessed if there was a relationship between the severity of inflammation at the site of intestinal mucosa in CD patients (using partial SES‐DC scoring system) and the levels of IL‐10, and we did not find any relationship for in IL‐10 mRNA nor protein expression. In fact, this would be expected for a cytokine that is immunoregulatory, not proinflammatory.

Altogether, these data demonstrated on the one hand that increased IL‐10, both at the gene and the protein level, are mainly related to intestinal inflammation regardless of its etiology (see summary of results in Table), and on the other, that location‐dependent differences also exist with an increase in IL‐10 protein levels in the ileum, both in healthy and inflamed mucosa (Table 1).

**Table 1:** Summary of results of the different Interleukin‐10 (IL‐10) determinations in Crohn's disease, ulcerative colitis and infectious colitis as compared to controls

IL‐10	Crohn's disease*		Ulcerative colitis*	Infectious colitis*
Intestinal Mucosa				
mRNA levels	↑		↑	=
Secreted protein	↑		↑	↑
Tissue protein Colon	=		=	↑
Tissue protein Ileum	↑		NA	NA
Serum	=		↑	=
Intestinal mucosa	Steroid‐dependent**	Steroid‐refractory**		
mRNA levels	↓	↓↓		
Secreted protein	=	=		
Tissue protein	=	=		
Serum	=	↑		

*Note*: *Samples obtained before treatment initiation. In Crohn's disease the results are also provided regarding steroid response; **in steroid‐dependent and steroid‐refractory compared to sensitive patients, one month after treatment initiation. (↑ increased, ↓ decreased, or = unchanged).

Abbreviations: mRNA, messenger RNA; NA, not applicable.

### 3.4 Mucosal IL‐10 gene expression levels and steroid response in CD

Our group previously demonstrated that high colonic IL‐10 mRNA predicted a good response to steroids in samples taken before steroid initiation, and these increased IL‐10 mRNA levels were maintained in follow‐up samples taken after steroid administration in steroid‐sensitive patients.^5^ Thus, with those promising results, we attempted to replicate them in a new cohort of patients and to evaluate the potential usefulness in clinical practice of the assessment of IL‐10 mRNA.

**Figure 3:**
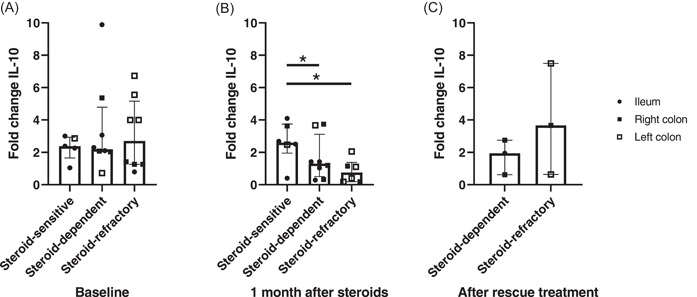
Interleukin‐10 gene (IL‐10) expression in inflamed mucosa of Crohn's disease according to steroid response at (A) baseline before treatment, (B) 1 month after steroid initiation and (C) after rescue treatment in steroid‐sensitive (*n* = 5), steroid‐dependent (*n* = 8), and steroid‐refractory patients (*n* = 8). Bar graph represents median values and whiskers above and below 5%−95% percentile. Kruskall−Wallis Test. *p < .05 versus steroid‐sensitive patients.

Disappointingly, IL‐10 mRNA values could not separate patients according to steroid response at baseline (*p* = .934) (Figure 3A). By contrast, in line with the previously published data, those patients with steroid refractoriness or dependency showed lower IL‐10 gene expression levels as compared to steroid‐sensitive patients, 1‐month after steroid treatment initiation (*p* = .027) (Figure 3B). It is noteworthy that mucosal samples of steroid‐dependent patients showed intermediate values between those of the steroid‐sensitive and steroid‐refractory. To determine whether IL‐10 changes were due to a reduction in expression in non‐responders or an increase in steroid‐sensitive patients, we compared baseline and after‐treatment levels with two independent calculations: change over the control group (Supporting Information: Figure 2A) or change over the baseline (Supporting Information: Figure 2B) sample of the same patient. The two measurements offered similar results, showing that nonresponsive patients suffered a dramatic reduction in their IL‐10 levels, whereas levels of the steroid‐sensitive patients remained with steady values of IL‐10 mRNA without changes related to treatment (Supporting Information: Figure 2A−B). Moreover, in those patients with steroid refractoriness or dependency, IL‐10 levels increased after achieving clinical remission with a rescue treatment (biologics and/or immunosuppressants). All of these findings (sustained Il‐10 levels in steroid‐sensitive patients, decrease in nonresponders and recover in responders to rescue therapy) suggest that appropriate IL‐10 levels are required to achieve and maintain clinical response, whereas reduced IL‐10 gene expression may lead to disabling CD if not reversed with appropriate treatment (Figure 3C and Supporting Information: Figure 2A−B).

As regards to the influence of steroid treatment in IL‐10 protein expression, we did not find any differences according to treatment response at baseline or poststeroid treatment levels. Differences between pre and posttreatment samples were not found either among the patients as a whole or in any of the response groups (Supporting Information: Figure 3). Hence, according to our results, the increased production of secreted IL‐10 seems to constitute a nonspecific feature of intestinal inflammation, irrespective of the type of disease, severity, or response to steroid treatment.

IL‐10 protein expression assessed with immunofluorescence did not show differences related to therapeutic response, either in terms of the MFI and LP/SD scores or the cell types responsible for IL‐10 synthesis (Supporting Information: Figure 4).

### 3.5 Peripheral serum IL‐10 levels

**Figure 4:**
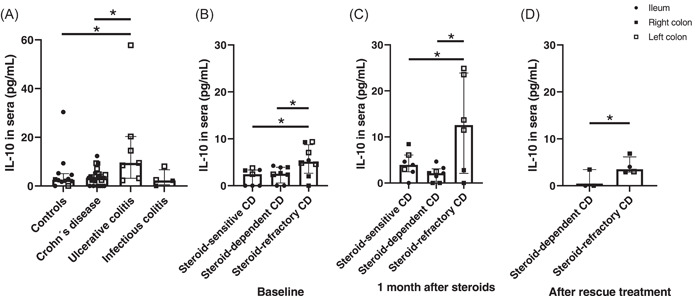
Interleukin‐10 (IL‐10) levels in peripheral blood of controls (*n* = 12), Crohn's disease (CD, *n* = 25), ulcerative colitis (*n* = 7), and infectious colitis (*n* = 4) patients (A). In CD patients, IL‐10 levels are provided at baseline before treatment (B), 1 month after steroid initiation (C), and after rescue treatment (for those nonresponders that required immunosuppressants or biologics) (D). Results are expressed as pg of protein per ml in sera. Bar graph represents median values and whiskers above and below 5%−95% percentile. Mann−Whitney *U* Test (A); Kruskall−Wallis Test (B, C, D). *p < .05.

Finally, we measured levels of IL‐10 in peripheral blood to determine whether there was a relationship between the intestinal and systemic compartments in terms of IL‐10 dynamics. In addition, in the event that there was any possible clinical application, this would be the easiest and cheapest measure to implement in every‐day practice and, unlike bowel measurements, it is not limited by compartment restrictions. Similarly, as previously reported,^18^ a significant increase in UC patients was found compared to controls (*p* = .028) and CD patients (*p* = .010), whereas no differences were found among CD, IC, and controls (Figure 4A).

Regarding the levels of IL‐10 related to steroid response in CD, significantly high levels in steroid‐refractory patients were found at baseline (before starting steroids) as compared to steroid‐sensitive (*p* = .029) and steroid‐dependent (*p* = .050) patients (Figure 4B). Moreover, this increase was even more pronounced 1 month after steroid initiation due to further increases in refractory patients (*p* < .05 vs. dependent and sensitive), (Figure 4C). The same differences were maintained after rescue therapy, even though values were lower than those obtained after 1 month of steroid treatment in steroid‐refractory patients (Figure 4D).

Table offers a summary of the levels of IL‐10 in intestinal mucosal and peripheral blood.

## 4 DISCUSSION

We previously demonstrated that high levels of mRNA‐IL‐10 in the intestinal mucosa was a good predictor of steroid response in CD. In addition, values for IL‐10 remained significantly higher under steroids in sensitive patients, revealing an improved diagnostic accuracy as steroid response predictor during the follow‐up (Sensitivity 100%; 100% Negative Predictive Value).^5^

In the present validation cohort, we reproduced the same results in the posttreatment sample (1 month after steroid initiation) but not at baseline. Patients with good response to steroids showed sustained high mucosal levels of mRNA IL‐10, whereas a significant decrease was observed in steroid‐dependent and refractory patients. Thus, in the present cohort, compared to the previous one, depletion of mRNA IL‐10 in steroid‐resistant patients occurred at a later stage of the disease flare‐up, arguing against its use as a predictive marker. It may be speculated that different clinical characteristics in the present CD population compared to the previous one account for this delayed IL‐10 mRNA reduction in nonresponders. In fact, the majority of patients in the present cohort were included at diagnosis, whereas in the previous one, only half of the patients had new‐onset CD. Although mRNA IL‐10 levels cannot be used at diagnosis to predict steroid response, this phenomenon is consistent and seems to have physiopathological relevance given that IL‐10 gene expression is restored in patients achieving remission with rescue therapy.

In spite of the reduced mucosal IL‐10 mRNA in nonresponsive CD patients, we could not find differences in the mucosal IL‐10 protein expression, with similar amounts of IL‐10 produced in culture explants irrespective of treatment response. We also did not find differences when analyzing tissue slides with IF or using user‐dependent score measurements of IL‐10 positive cells (in SD or LP), either with objective quantification of the MFI and the percentage of tissue surface covered by IL‐10. We could detect both subepithelial dendritic cell type morphology and also LP lymphocyte positive cells staining for IL‐10. In fact, a good number of cell populations involved in adaptive immune response (Th1, Th2, Th17, Tregs, and B cells) express IL‐10.^1^ Which cell types express IL‐10 in healthy and inflamed intestine is not fully defined in humans, therefore future studies evaluating co‐localization of cell markers and IL‐10 staining would be needed to shred light in this matter. The reason why we did not find differences between groups in terms of IL‐10 synthesis is probably the pleiotropic capacity of this ubiquitous cytokine that is needed to maintain the intestine healthy, downregulating chronic and acute inflammation and inducing mucosal repair.^1^, ^19^

By contrast, refractory CD patients, but not all the CD cohort, showed elevated IL‐10 serum levels compared to controls. Thus, serum levels of IL‐10 may be useful to discriminate CD patients with steroid resistance even before the start of treatment. In addition, this pattern is maintained throughout the course of the disease, even when remission is achieved after using rescue therapy. It could be hypothesized that reduced IL‐10 mRNA in the intestinal mucosa of these patients is the result of increased local and systemic requirements of Il‐10 to dampen the inflammatory process, as previously suggested.^20^ At any rate, the systemic increase of IL‐10 does not seem to be related to the disease itself, nor to disease severity, as previously described,^21^ but rather to a refractory condition. It is unknown if the particular IL‐10 local and systemic dynamics related to refractory CD are due to the presence of particular IL‐10 haplotypes. Genetic variations may influence not only disease development but also the appearance of certain prognostic patterns and therapeutic responses.^22^ For example, a relationship between higher producing IL10‐1082G and TNFa857C alleles with complicated CD has previously been found in an Australian cohort,^23^ and high levels of circulating IL‐10 caused by different haplotypes of the IL‐10 promoter region were related to fatal outcome in menigoccocal disease.^24^ From a clinical point of view, circulating levels of IL‐10 have been included in multimarker predictive models of cardiovascular disease^25^ and also positively associated with risk of cardiovascular events.^26^ Disappointingly, from the results of our study the usefulness at the clinical level of this measurement seems limited to refractory CD patients, since it was unable to identify those patients with incomplete response (steroid‐dependent patients). The major limitation of our study is the small sample size evaluated. Therefore, our findings deserve to be confirmed in further studies with a larger number of CD patients with different patterns of therapeutic response.

We also found increased levels of IL‐10 mRNA in all the forms of colonic and ileal intestinal inflammation, as previously found in IBD^27^, ^28^ and microscopic colitis.^28^ In addition, increased IL‐10 mRNA levels in CD and UC paralleled increases in IL‐10 protein expression in culture supernatant of mucosal explants. By contrast, IL‐10 detection in tissue homogenates showed a regional differentiation, with increased production in ileum compared to colon. IL‐10 protein in culture supernatant represents the balance between synthesis and degradation of the protein excreted in the culture milieu, whereas IL‐10 in tissue homogenates reflects all the intra‐ and extracellular protein at a specific time. The regional immunological differences associated with particular intestinal compartments highlights the need for a prior functional and morphological assessment in the intestinal location that we are investigating in both health and disease.^10^

This study also reveals a dynamic link between IL‐10 gene and protein expression that is key to understanding its pathophysiological effect. In this sense it is well known that increased gene expression of a cytokine does not necessarily imply an increase in its protein level. This may be for several reasons such as rapid degradation and consumption by immune cells, suppression at a posttranscriptional level of gene expression induced either by regulatory cells such as double negative T cells or Treg cells, or microRNA regulation.^28^, ^29^, ^30^, ^31^

In summary, we have confirmed, in an independent CD cohort, a particular mucosal IL‐10 mRNA pattern related to steroid‐resistant CD. In addition, high levels of circulating IL‐10 were found in this population, which may have potential clinical applications. These results will serve as a useful guide towards improved understanding of treatment failure in CD. Further assessment with a larger sample size with clinical and mechanistic studies to investigate the role of IL‐10 in treatment resistance is warranted.

## AUTHOR CONTRIBUTIONS

Anna Carrasco designed and performed laboratory experiments, performed statistical analysis, prepared figures, and drafted the manuscript. Maria Esteve conceived and designed the study, revised, edited, and finalized the manuscript, coordinated the research group, and directed the execution of the study. Fernando Fernández‐Bañares revised, edited, and finalized the manuscript. Eva Tristán performed laboratory experiments and statistical analysis, prepared figures, and drafted the manuscript. Montserrat Aceituno, Albert Martín‐Cardona, Yamile Zabana, Carme Loras, and Xavier Andújar, were in charge of CD patients and collected data related to treatment response. Violeta Lorén, Josep Manyé, Lourdes Fluvià, and José María Hernández performed laboratory experiments. Antonio Salas performed the pathological examination of tissue samples. All authors read and approved the final version of the manuscript.

## CONFLICT OF INTEREST

The authors declare no conflict of interest.

## ETHICS STATMENT

The ethical committee of our institution approved this study (Ethical committee name: Comité Ético de Investigación con Medicamentos. Approval number: Acta 02/2010). Signed informed consent was obtained from all subjects before sample collection and all the experiments were conducted following the principles set out in the WMA Declaration of Helsinki and the Department of Health and Human Services Belmont Report.

## Supporting information

Supporting information.

Supporting information.

Supporting information.

Supporting information.

Supporting information.

## Data Availability

The authors declare that all other relevant data generated or analyzed during this study are included in the article, the extended data file, or the supporting information files. Materials, protocols and images are available from the corresponding author on reasonable request.
