# Supplementary material for: Mucosal Interleukin‐10 depletion in steroid‐refractory Crohn's disease patients
Source: Immun Inflamm Dis. 2022 Sep 27;10(10):e710. doi: 10.1002/iid3.710 (PMC9514060; doi:10.1002/iid3.710)
Supplement: Supplementary file 1 — Supporting information. [file IID3-10-e710-s002.pdf]

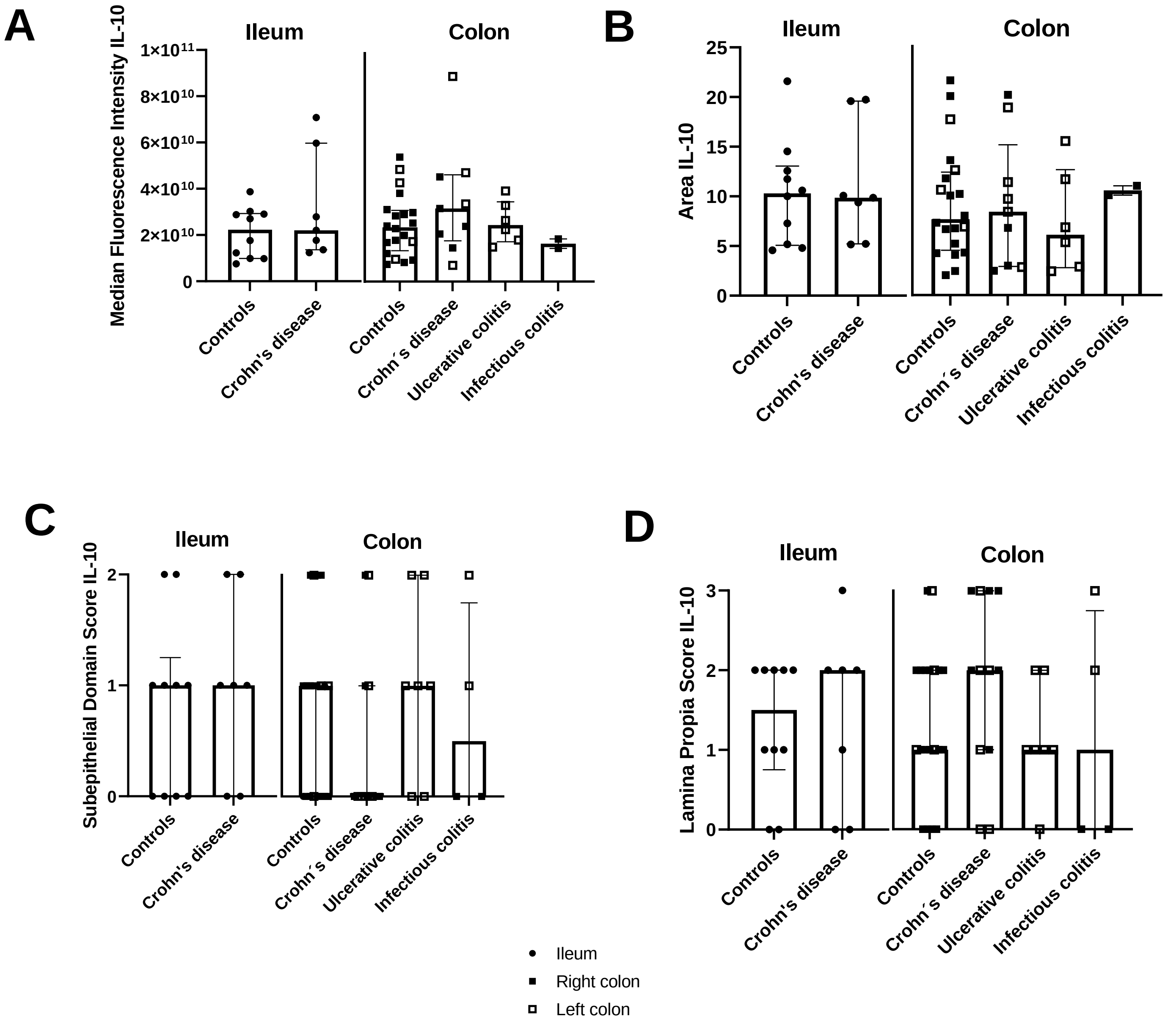

Supplementary Figure 1. Interleukin-10 (IL-10) protein expression assessed with immunofluorescence in different intestinal regions (ileal and colon) of healthy subjects (n=10 paired samples) and inflamed mucosa of Crohn’s disease (n= 16), ulcerative colitis (n=7), and infectious colitis (n=4) patients. Median fluorescence intensity (A), area (B), subepithelial domain (C), and lamina propria (D) expression. Bar graph represents median values and whiskers above and below 5-95% percentile. Kruskall-Wallis Test.
