# Supplementary material for: Mucosal Interleukin‐10 depletion in steroid‐refractory Crohn's disease patients
Source: Immun Inflamm Dis. 2022 Sep 27;10(10):e710. doi: 10.1002/iid3.710 (PMC9514060; doi:10.1002/iid3.710)
Supplement: Supplementary file 2 — Supporting information. [file IID3-10-e710-s003.pdf]

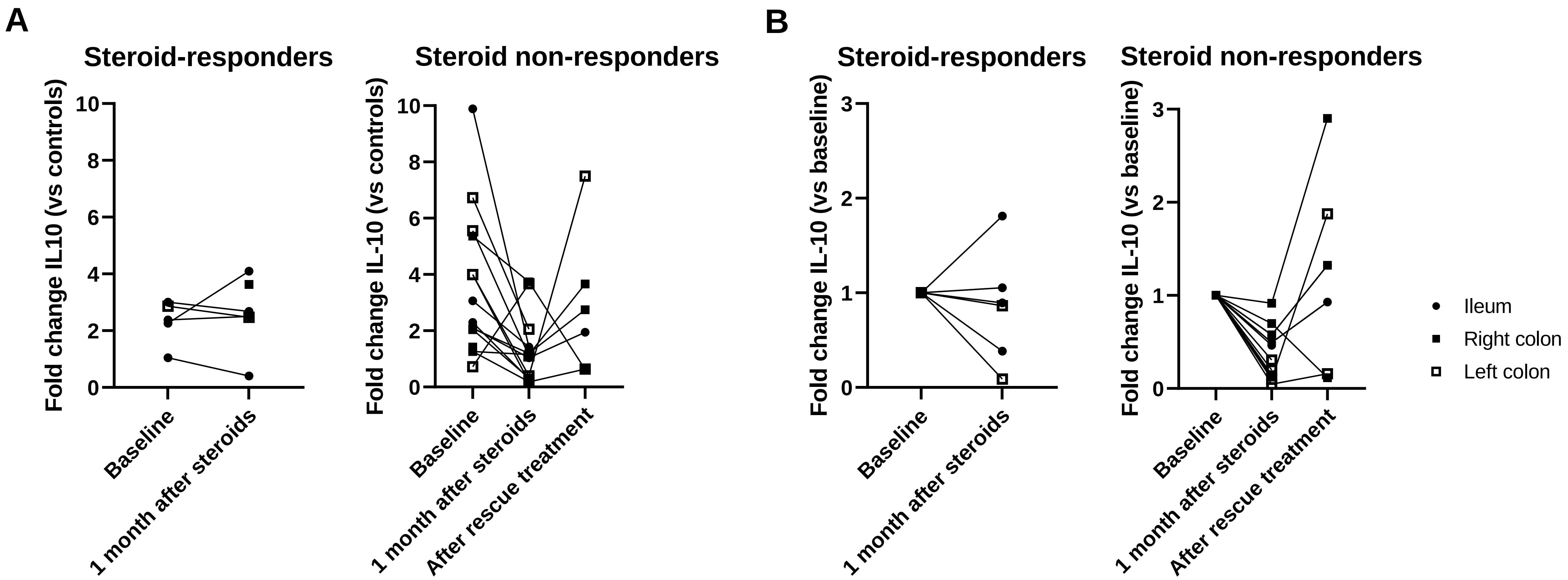

Supplementary Figure 2. Evolution of Interleukin-10 (IL-10) gene expression related to steroid response (steroid sensitive patients n=5; steroid non-responders n= 14) in Crohn's disease inflamed mucosa. Steroid non-responders include both refractory and dependent patients. Results are expressed as fold change over the values of controls (A) or over the baseline sample of the same patient (B). Measurements were made at baseline, 1 month after steroid initiation, and after rescue treatment (for those non-responders that received immunosuppressants or biologics). Wilcoxon rank test.
