# Supplementary material for: Mucosal Interleukin‐10 depletion in steroid‐refractory Crohn's disease patients
Source: Immun Inflamm Dis. 2022 Sep 27;10(10):e710. doi: 10.1002/iid3.710 (PMC9514060; doi:10.1002/iid3.710)
Supplement: Supplementary file 3 — Supporting information. [file IID3-10-e710-s004.pdf]

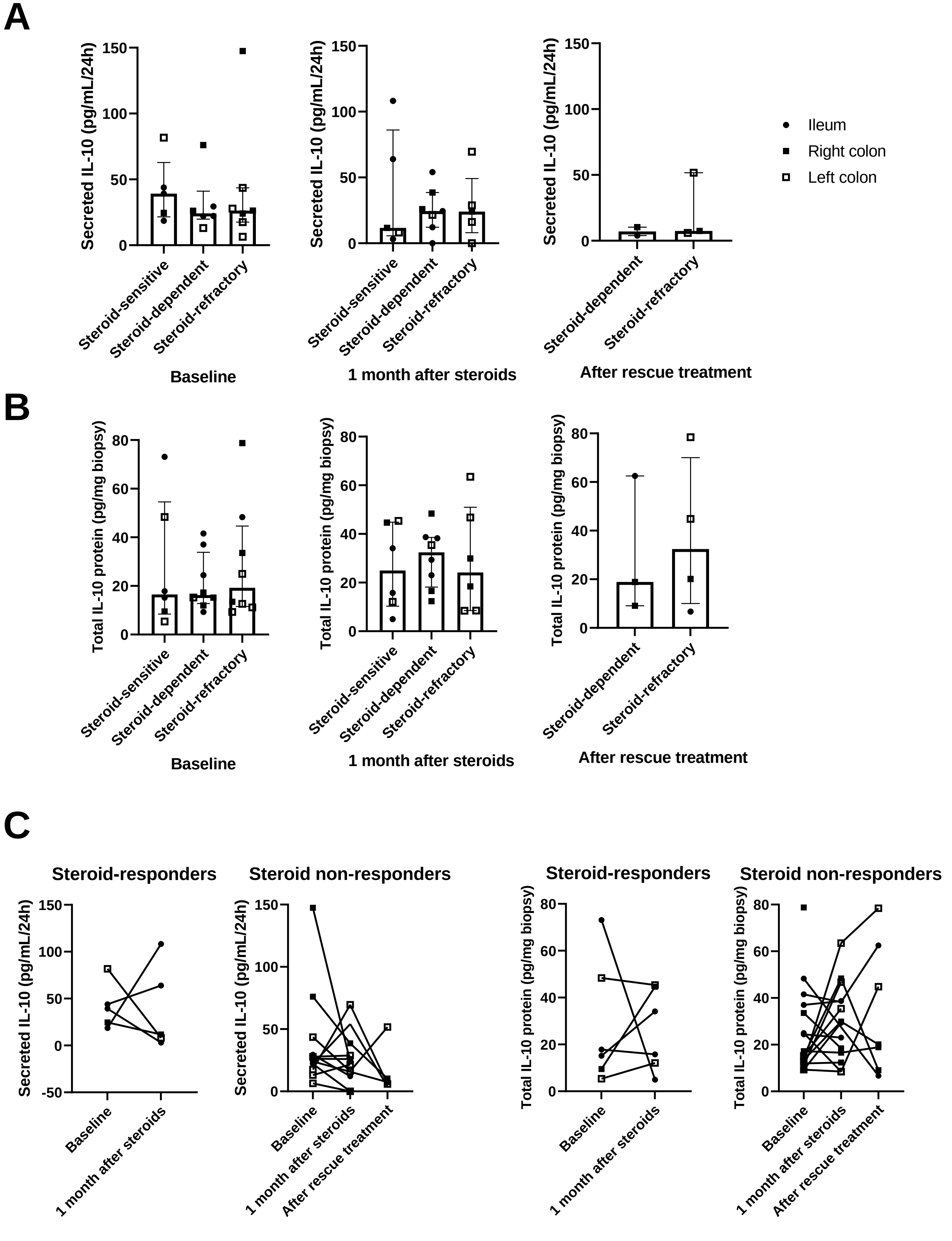

Supplementary Figure 3. Protein levels of Interleukin-10 (IL-10) in inflamed mucosa of Crohn's disease related to steroid response (steroid sensitive n=5; steroid dependent n=8 and steroid refractory n=6 patients) in secreted (A) and total IL-10 protein (B). C: Evolution of secreted and total IL-10 protein expression in inflamed mucosa of Crohn's disease in steroid responders and steroid non-responders (refractory and dependent). Results are expressed as pg of protein per mL in 24-hour culture and in biopsy homogenate. Kruskall-Wallis Test (A, B). Friedman Test (C).
