# Supplementary material for: Mucosal Interleukin‐10 depletion in steroid‐refractory Crohn's disease patients
Source: Immun Inflamm Dis. 2022 Sep 27;10(10):e710. doi: 10.1002/iid3.710 (PMC9514060; doi:10.1002/iid3.710)
Supplement: Supplementary file 4 — Supporting information. [file IID3-10-e710-s001.pdf]

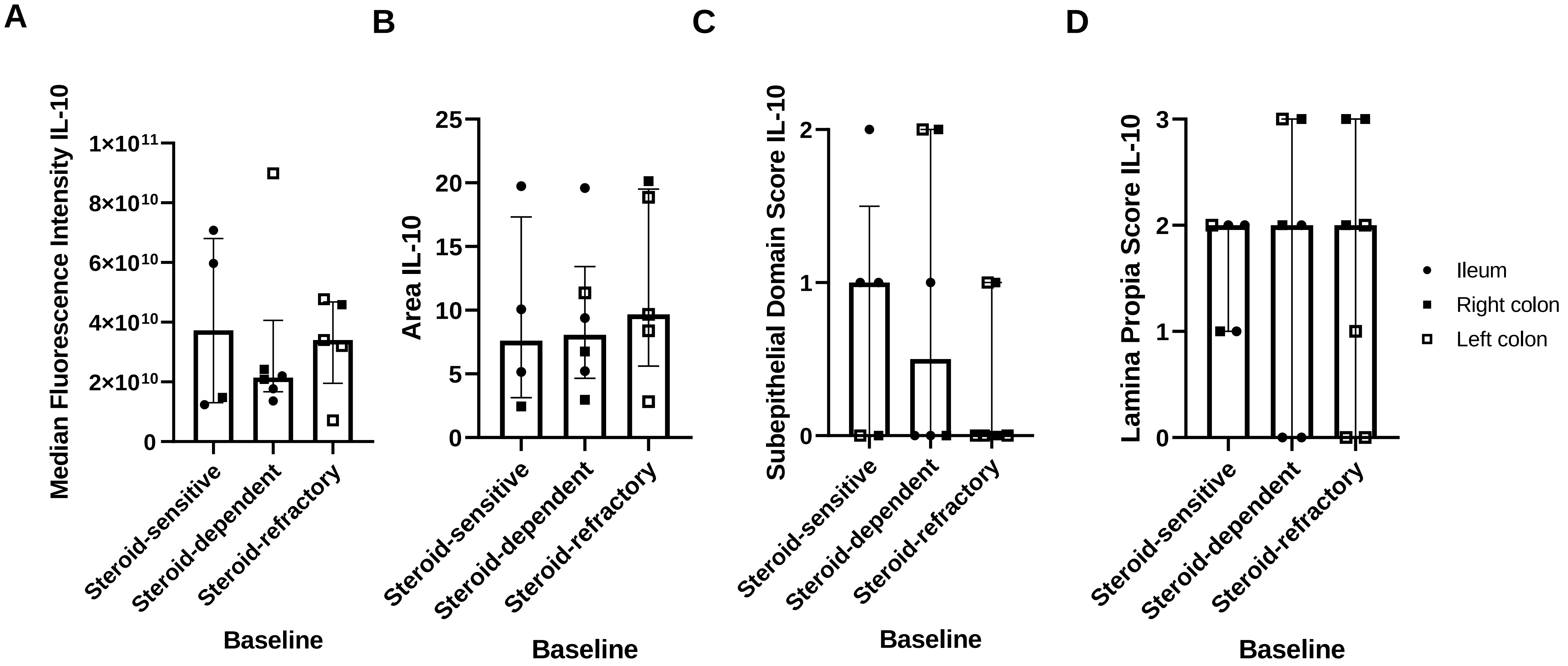

Supplementary Figure 4. Interleukin-10 (IL-10) expression assessed with immunofluorescence in inflamed mucosa of Crohn’s disease patients related to therapeutic response (steroid sensitive n=5; steroid dependent n=6 and steroid refractory n=7 patients) at baseline. Median fluorescence intensity (A), area (B), subepithelial domain (C), and lamina propria (D).
