# Supplementary figures and images for: Mucosal Interleukin‐10 depletion in steroid‐refractory Crohn's disease patients
Source: Immun Inflamm Dis. 2022 Sep 27;10(10):e710. doi: 10.1002/iid3.710 (PMC9514060; doi:10.1002/iid3.710)

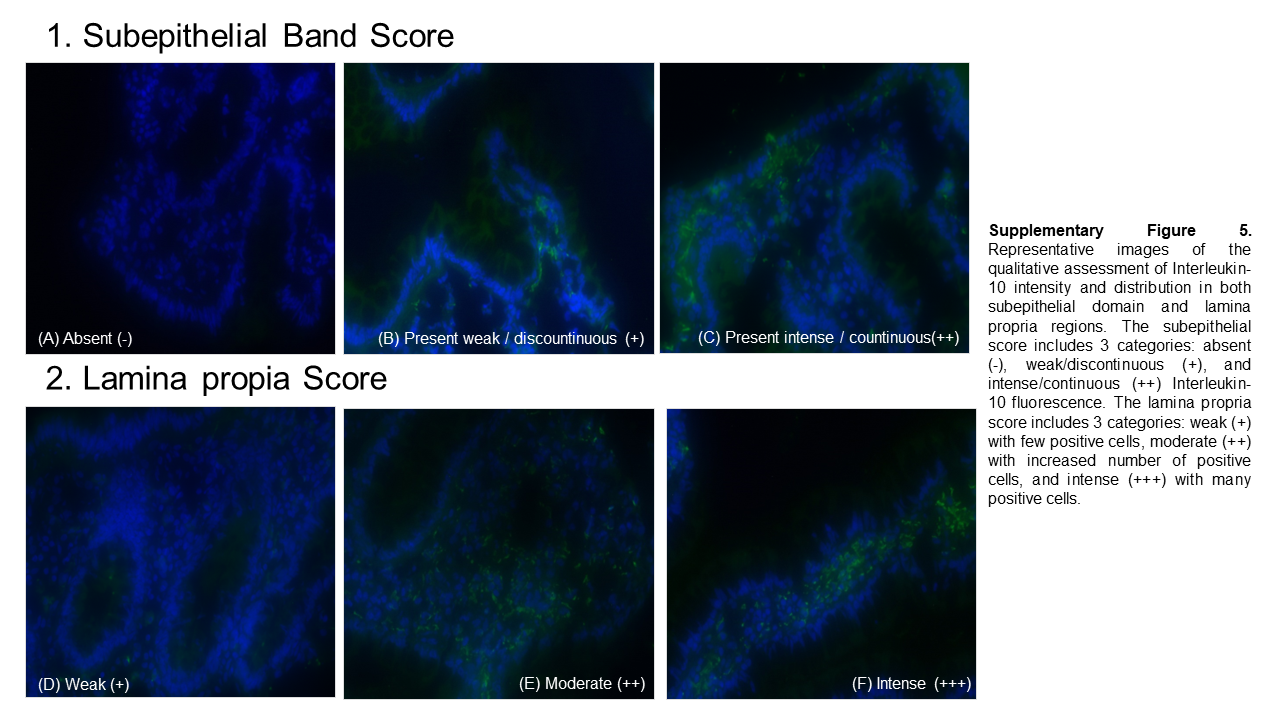

Supplement: Supplementary file 5 — Supporting information. [file IID3-10-e710-s005.tif]
